# Supplementary material for: Phosphorylation of human glioma-associated oncogene 1 on Ser937 regulates Sonic Hedgehog signaling in medulloblastoma
Source: Nat Commun. 2024 Feb 2;15:987. doi: 10.1038/s41467-024-45315-x (PMC10837140; doi:10.1038/s41467-024-45315-x)

Supplementary Information

Phosphorylation of human glioma-associated oncogene 1 on Ser937 regulates Sonic Hedgehog signaling in medulloblastoma

Ling-Hui Zeng[1,*,†], Chao Tang[2,3,*], Minli Yao[2,4,*], Qiangqiang He[2,4], Meiyu Qv[1,2], Qianlei Ren[1], Yana Xu[2,4], Tingyu Shen[2,4], Weizhong Gu[3], Chengyun Xu[1,2,3], Chaochun Zou[3], Xing Ji[1,2], Ximei Wu[2,4,†], Jirong Wang[5,†]

[1]Department of Pharmacology, Hangzhou City University School of Medicine, Hangzhou, 310015, China;

[2]Department of Pharmacology, Zhejiang University School of Medicine, Hangzhou, 310058, China;

[3]National Clinical Research Center for Child Health, the Children's Hospital of Zhejiang University School of Medicine, Hangzhou 310053, China;

[4]Department of Orthopaedics, the Affiliated Sir Run Run Shaw Hospital, Zhejiang University School of Medicine, Hangzhou, 310016, China;

[5]Department of Geriatrics, Zhejiang Hospital, Hangzhou, 310030, China.

[†]Corresponding author. Email: zenglh@zucc.edu.cn; xiwu@zju.edu.cn; wangjr@zju.edu.cn

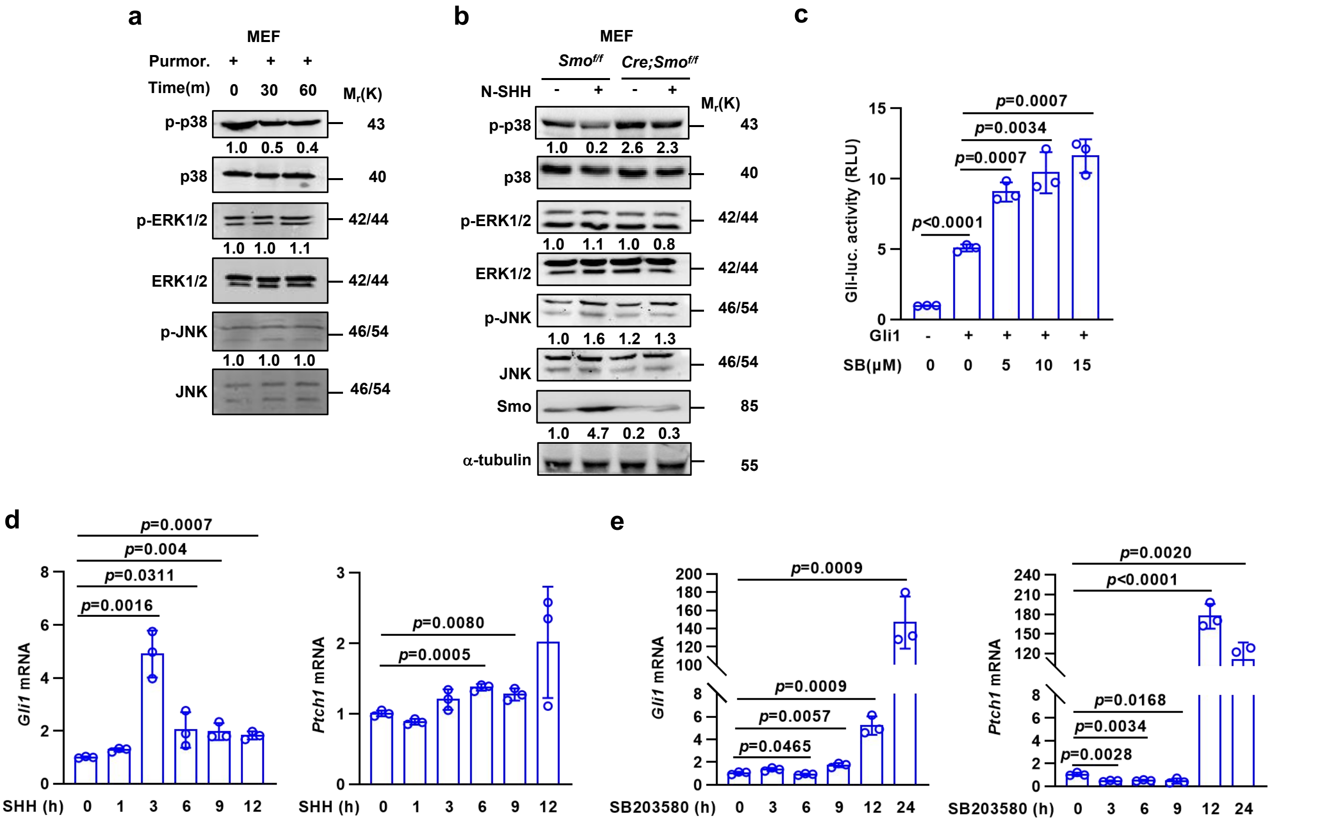

Supplementary Figure 1. Activation of SHH signaling inactivates p38 to induce the transcriptional output.
a and **b** Western blot analyses in MEFs treated with purmorphamine or N-SHH at 2 μM or 100 ng/ml for the indicated times or 60 min, respectively. **c** Gli-luciferase reporter assays in C3H10T1/2 cells transfected with Gli1 and then treated with the indicated dosages of SB203580 for 48 h (*n*=3 independent experiments). **d** and **e** Quantitative RT-PCR analysis for *Gli1* and *Ptch1* in C3H10T1/2 cells treated with N-SHH at 100 ng/ml or SB203580 at 10 μM for the indicated times (*n*=3 independent experiments). Data were presented as mean ±sd, two-tailed Student's *t*-test. A representative example of three replicates is shown for **a** and **b**. Source data are provided as a Source Data file.

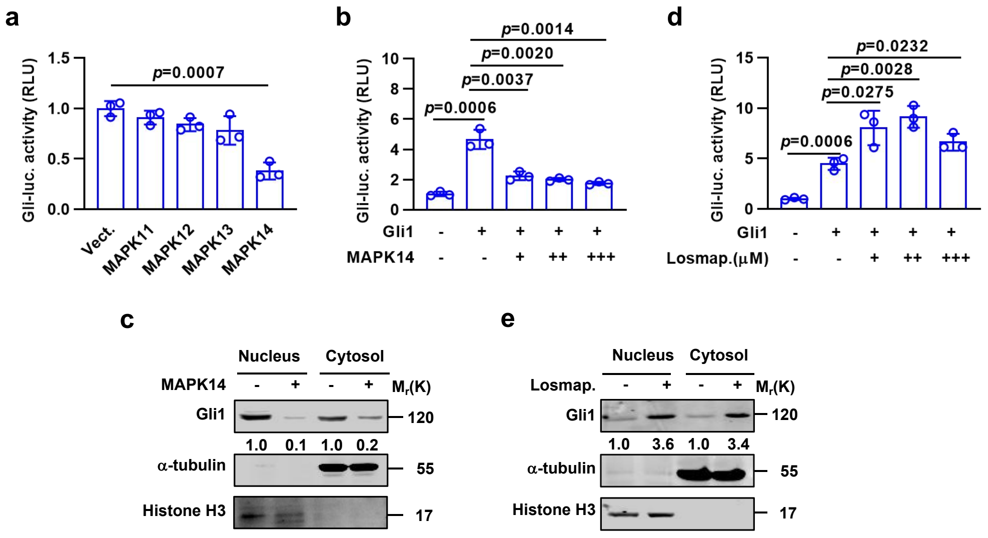

Supplementary Figure 2. P38α negates Gli1 levels and transcriptional activity.

a-c Gli-luciferase reporter and western blot analyses in C3H10T1/2 cells at 24 h post transfection with MAPK11, MAPK12, MAPK13 or MAPK14 in the presence or absence of Gli1 (n=3 independent experiments). **d** and **e** Gli-luciferase reporter and western blot analyses in C3H10T1/2 cells transfected with or without Gli1 and treated with or without losmapimod at 10 μM for 48 h (n=3 independent experiments). Data were presented as mean ±sd, two-tailed Student's t-test for **a**, **b**, and **d**. A representative example of three replicates is shown for **c** and **e**. Source data are provided as a Source Data file.

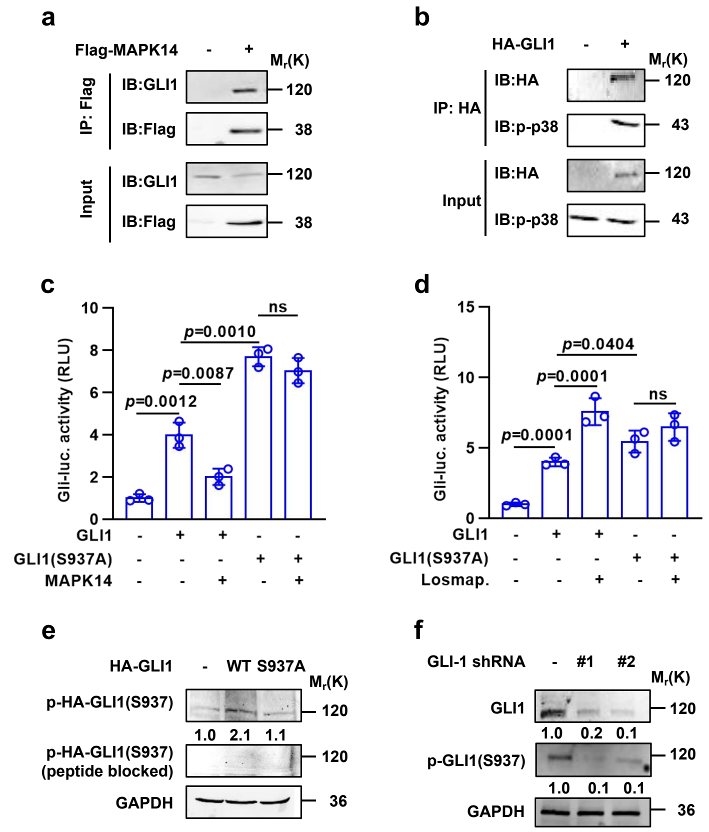

Supplementary Figure 3. Regulation of GLI1 phosphorylation by p38.

a and **b** Co-immunoprecipitation experiments by using a Flag or HA antibody in 293T cells transfected with Flag-MAPK14 or HA-GLI1, respectively. **c** and **d** Gli-luciferase reporter assays in 293T cells transfected with the indicated constructs or transfected with the indicated constructs and treated with losmapimod at 10 µM for 48 h (n=3 independent experiments). **e** Western blot analysis of phospho-S937-GLI1 and GLI1 in 293T cells transfected with HA-GLI1(WT) or HA-GLI1(S937A) and immunoblotted with a phospho-S937-GLI1 antibody in combination with or without a phospho-S937-GLI1 neutralizing peptide. **f** Western blot analysis of phospho-S937-GLI1 and GLI1 in 293T cells transfected with scramble- or GLI1-shRNA (#1 or #2). Data were presented as mean ±sd, two-tailed Student's t-test for **c** and **d**. A representative example of two replicates is shown for **a**, **b**, **e**, and **f**. Source data are provided as a Source Data file.

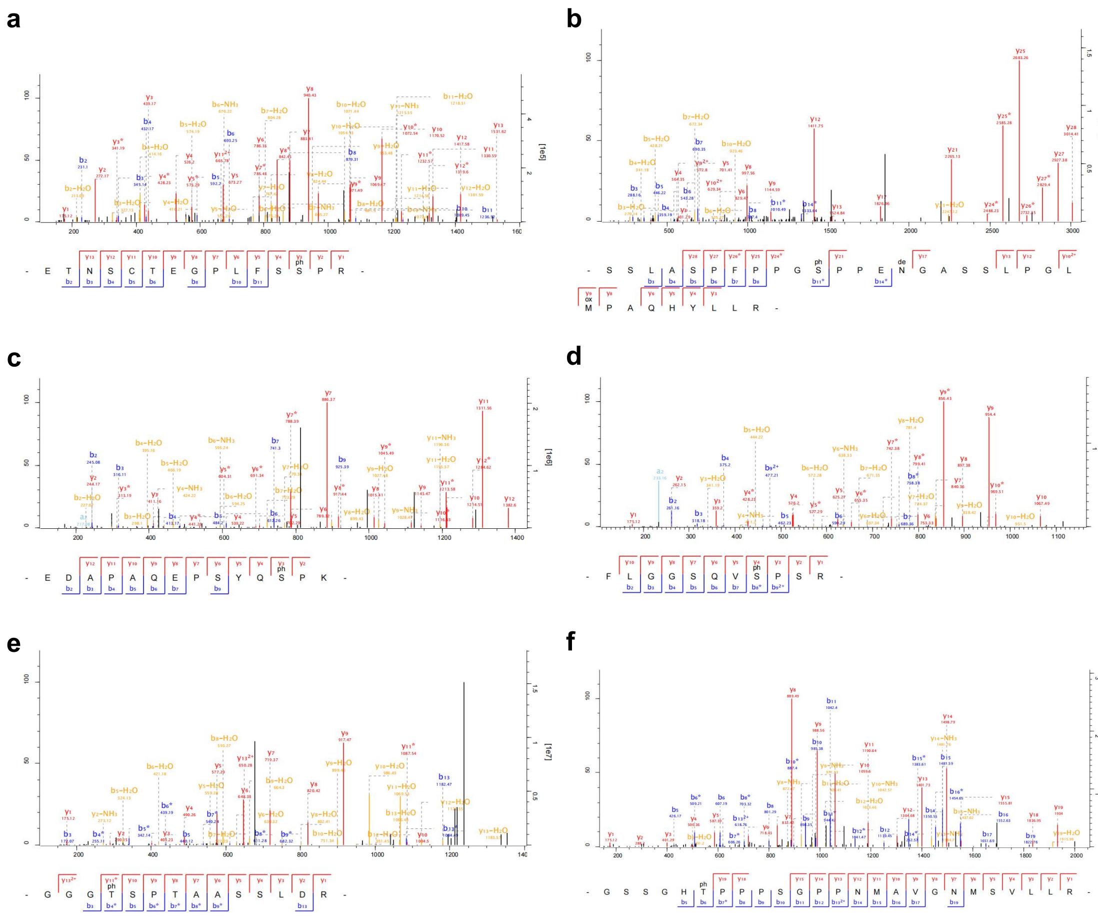

Supplementary Figure 4. Potential human GLI1 phosphorylation sites *in vivo*.

Potential human GLI1 phosphorylation sites S70 (a), S569 (b), S927 (c), S937 (d) and T601 (e), T1074 (f) *in vivo*. GLI1 was purified from Daoy cells and analyzed by MS. Source data are provided as a Source Data file.

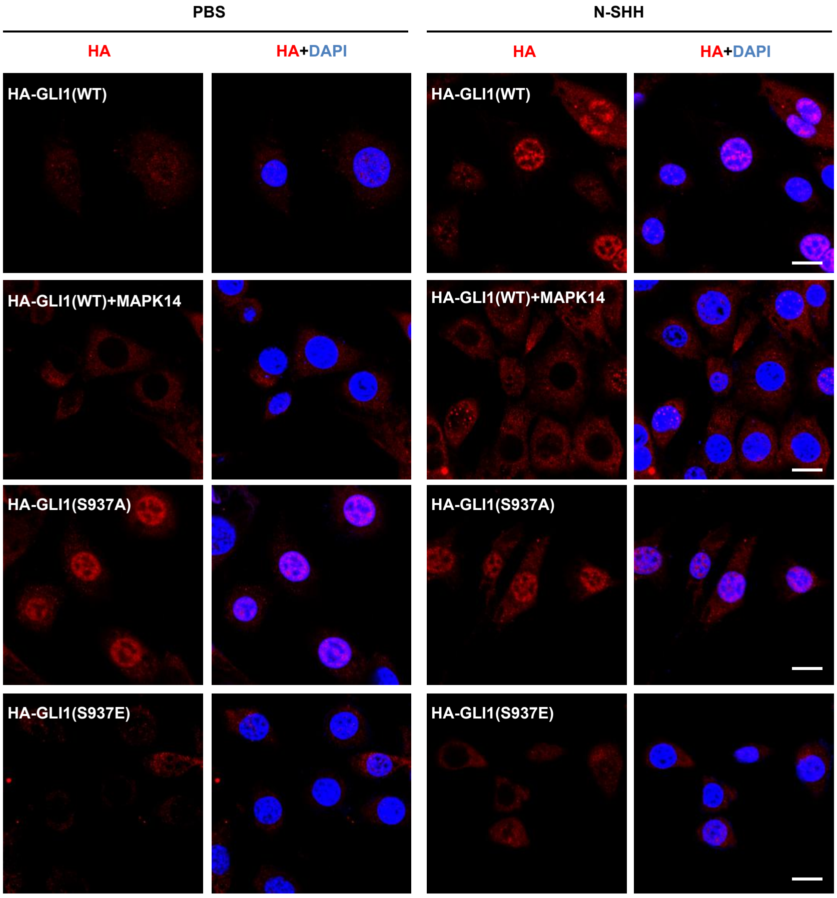

Supplementary Figure 5. Cytosolic and nuclear distribution of HA-GLI1 variants.

Immunofluorescence staining for HA in C3H10T1/2 cells transfected with indicated HA-tagged plasmids and treated with or without N-SHH at 100 ng/ml for 30 min. Nuclei were counterstained with DAPI. Bar, 5 μm. A representative example of three replicates is show.

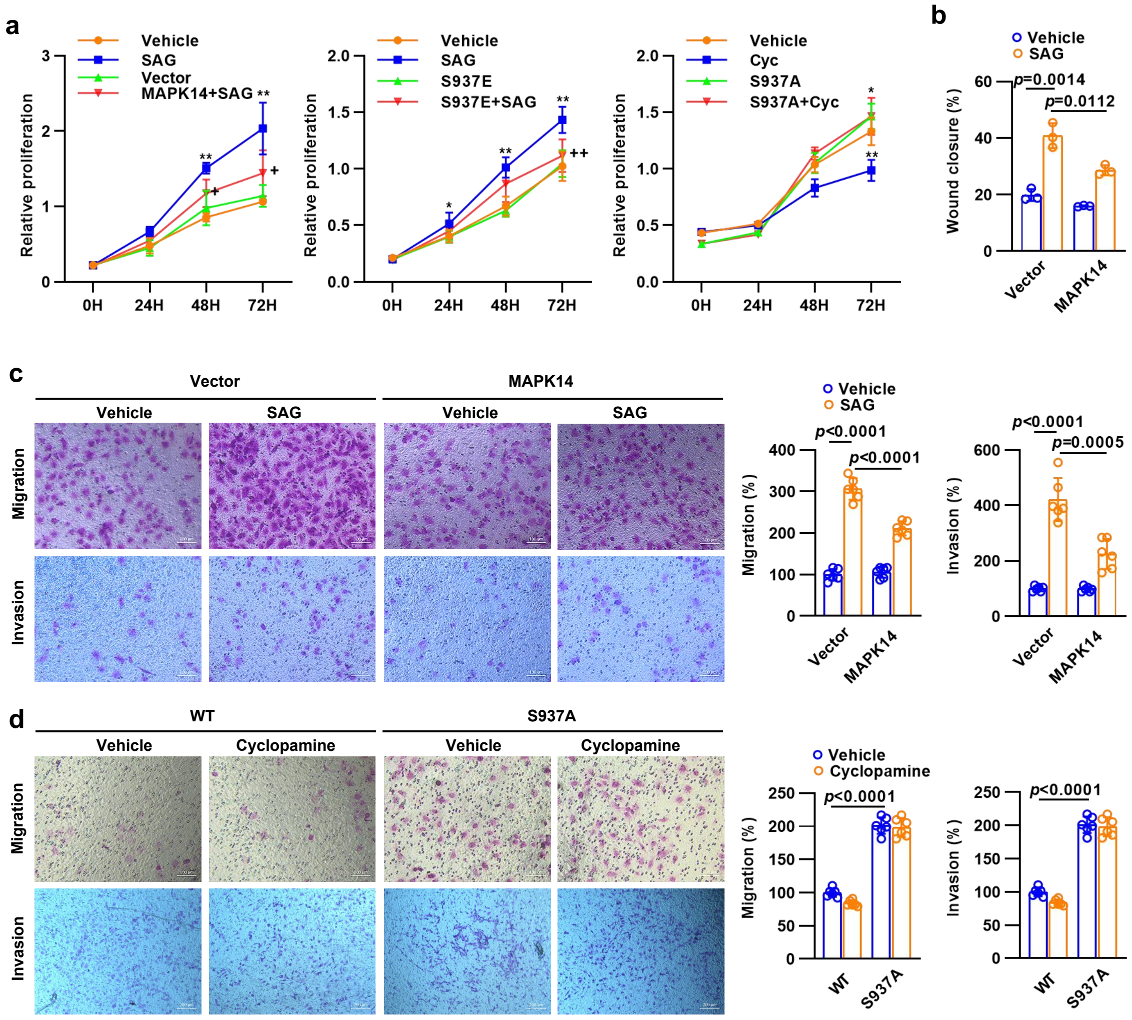

Supplementary Figure 6. p38α inhibits the invasive and metastatic capacities of MB cells.

a CCK-8 cell proliferation assay was performed on Daoy cells transfected with GLI1 mutant and cultured with SAG or Cyclopamine (*n*=6 independent experiments). **b** Wound-healing assays of Daoy cells transfected with MAPK14 and treated with SAG for 48 h (*n*=3 independent experiments). **c** and **d** Transwell assays showing the migratory and invasive abilities of Daoy cells transfected with MAPK14 or GLI1 mutant and cultured with SAG or Cyclopamine for 24 h (*n*=6 independent experiments). Data were presented as mean ±sd, two-tailed Student's *t*-test for **b**, **c** and **d**. Source data are provided as a Source Data file.

Supplementary Table 1. List of primers used

Gene name	Primer sequence	Products (bp)
Gli1	5’-CACGCATCCCGAGCACC-3’	140
	5’-GTTCCCTCTACCACGCAGAC-3’	
Ptch1	5’-CATGCCAGAGACCAGGCTGA-3’	98
	5’-GTCTCGTAGGCCGTTGAGGTAGAA-3’	
GAPDH	5’-ACCCAGAAGACTGTGGATGG-3’	171
	5’-CACATTGGGGGTAGGAACAC-3’	

FigS1A

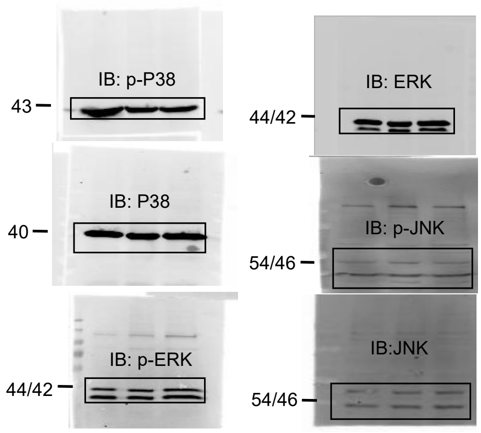

FigS1B

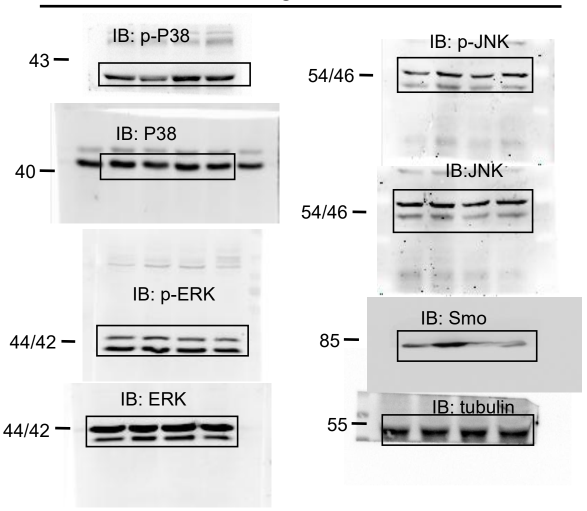

FigS2C

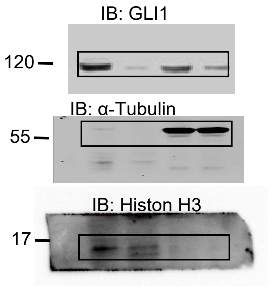

FigS2E

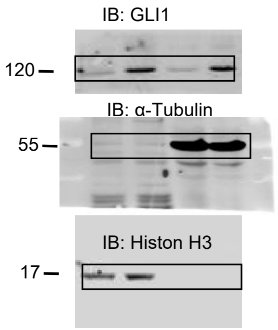

FigS3A

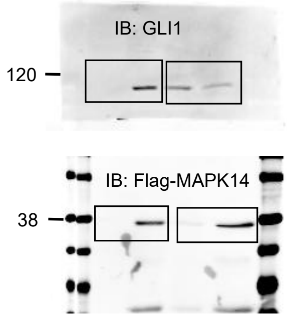

FigS3B

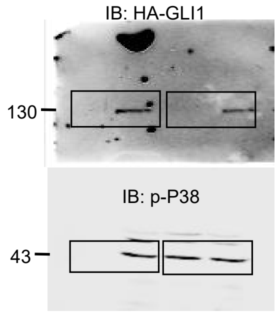

FigS3E

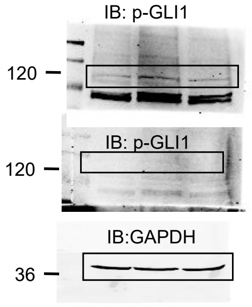

FigS3F

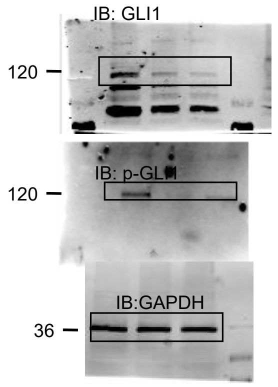

Supplement: Supplementary file 1 — Supplementary information [file 41467_2024_45315_MOESM1_ESM.pdf]
