# Supplementary material for: Phosphorylation of human glioma-associated oncogene 1 on Ser937 regulates Sonic Hedgehog signaling in medulloblastoma
Source: Nat Commun. 2024 Feb 2;15:987. doi: 10.1038/s41467-024-45315-x (PMC10837140; doi:10.1038/s41467-024-45315-x)
Supplement: Supplementary file 2 — Reporting Summary [file 41467_2024_45315_MOESM2_ESM.pdf]

nature portfolio

Corresponding author(s): Ximei Wu

Last updated by author(s): Dec 18, 2023

Reporting Summary

Nature Portfolio wishes to improve the reproducibility of the work that we publish. This form provides structure for consistency and transparency in reporting. For further information on Nature Portfolio policies, see our Editorial Policies and the Editorial Policy Checklist.

Statistics

For all statistical analyses, confirm that the following items are present in the figure legend, table legend, main text, or Methods section.

n/a	Confirmed	
☐	☒	The exact sample size (*n*) for each experimental group/condition, given as a discrete number and unit of measurement
☐	☒	A statement on whether measurements were taken from distinct samples or whether the same sample was measured repeatedly
☐	☒	The statistical test(s) used AND whether they are one- or two-sided<br>*Only common tests should be described solely by name; describe more complex techniques in the Methods section.*
☒	☐	A description of all covariates tested
☒	☐	A description of any assumptions or corrections, such as tests of normality and adjustment for multiple comparisons
☐	☒	A full description of the statistical parameters including central tendency (e.g. means) or other basic estimates (e.g. regression coefficient) AND variation (e.g. standard deviation) or associated estimates of uncertainty (e.g. confidence intervals)
☐	☒	For null hypothesis testing, the test statistic (e.g. *F*, *t*, *r*) with confidence intervals, effect sizes, degrees of freedom and *P* value noted<br>*Give P values as exact values whenever suitable.*
☒	☐	For Bayesian analysis, information on the choice of priors and Markov chain Monte Carlo settings
☒	☐	For hierarchical and complex designs, identification of the appropriate level for tests and full reporting of outcomes
☐	☒	Estimates of effect sizes (e.g. Cohen's *d*, Pearson's *r*), indicating how they were calculated

Our web collection on statistics for biologists contains articles on many of the points above.

Software and code

Policy information about availability of computer code

Data collection	Immunoblots were imaged using Bio-Rad Chemi-Doc Imaging system and Odyssey system, Image data was collected with Olympus fluoview FV 1200 and Zeiss LSM900. qPCR was performed on CFX-Touch 96. Dual-luciferase reporter assays were performed on Spark.
Data analysis	Quantification of Western blot was performed by ImageJ2X. Semi-quantitative analysis of the fluorescence and IHC signals was performed using ZEN 3.0 system. Statistics were calculated by the Micro soft Excel, IBM SPSS 13.0J and GraphPad Prim 8.

For manuscripts utilizing custom algorithms or software that are central to the research but not yet described in published literature, software must be made available to editors and reviewers. We strongly encourage code deposition in a community repository (e.g. GitHub). See the Nature Portfolio guidelines for submitting code & software for further information.

Data

Policy information about availability of data

All manuscripts must include a data availability statement. This statement should provide the following information, where applicable:

- Accession codes, unique identifiers, or web links for publicly available datasets
- A description of any restrictions on data availability
- For clinical datasets or third party data, please ensure that the statement adheres to our policy

The proteomics and IP-MS publicly available data used in this study have been deposited to the ProteomeXchange Consortium via the iProX partner repository with the dataset identifier PXD038222 and PXD046374. Source data are provided with this paper. All remaining data is available in the Article, Supplementary and Source Data files.

Research involving human participants, their data, or biological material

Policy information about studies with human participants or human data. See also policy information about sex, gender (identity/presentation), and sexual orientation and race, ethnicity and racism.

Reporting on sex and gender	Pathology slides of MB patients were obtained from both sexes and sex was not considered in the study design. Sex was determined based on self-report and findings were not categorized by sex or gender.
Reporting on race, ethnicity, or other socially relevant groupings	No reporting on race, ethnicity, or other socially relevant groupings.
Population characteristics	Among the 46 MB donors, all were children including 26 MBSHH who underwent surgery at hospital with an age range of 1-12 years.
Recruitment	All tissue sections used in this study were obtained from the Department of Pathology of Children's Hospital of Zhejiang University School of Medicine.
Ethics oversight	This study was approved by the Ethics Committee of the Children's Hospital of Zhejiang University School of Medicine (2023-IRB-0233-P-01).

Note that full information on the approval of the study protocol must also be provided in the manuscript.

Field-specific reporting

Please select the one below that is the best fit for your research. If you are not sure, read the appropriate sections before making your selection.

[x] Life sciences [ ] Behavioural & social sciences [ ] Ecological, evolutionary & environmental sciences

For a reference copy of the document with all sections, see nature.com/documents/nr-reporting-summary-flat.pdf

Life sciences study design

All studies must disclose on these points even when the disclosure is negative.

Sample size	No sample size calculation was performed. Samples sizes were determined based on minimum number of replicate to achieve statistical power. The cell samples were collected and performed with at least three independent experiments. The samples from animal were collected from three mice every group and performed with three independent experiments. A sample size of 119 or 212 mice was chosen for survival analysis. A sample size of 46 MB patients specimens was chosen in this study. This sample size is sufficient for significant statistical difference of clinicopathological characteristics. The exact size was described in the figure or the legend. For animal experiments, we choose same age of control and experiment littermate mice. For the cellular experiments, we count the cell number and use the same amount of control and experiment cells to do further experimetns.
Data exclusions	No data were excluded in the analysis in this study.
Replication	All experimental findings described in this study were repeated at least three times and got the consistent results before make relative conclusions.
Randomization	The samples/organisms/participants were randomly allocated into experimental group.
Blinding	All the authors involved in the experiments in this study were blinded to group allocation during data collection and/or analysis.

Reporting for specific materials, systems and methods

We require information from authors about some types of materials, experimental systems and methods used in many studies. Here, indicate whether each material, system or method listed is relevant to your study. If you are not sure if a list item applies to your research, read the appropriate section before selecting a response.

Materials & experimental systems

n/a	Involved in the study
☐	☒ Antibodies
☐	☒ Eukaryotic cell lines
☒	☐ Palaeontology and archaeology
☐	☒ Animals and other organisms
☒	☐ Clinical data
☒	☐ Dual use research of concern
☒	☐ Plants

Methods

n/a	Involved in the study
☒	☐ ChIP-seq
☒	☐ Flow cytometry
☒	☐ MRI-based neuroimaging

Antibodies

Antibodies used

JNK (Cell Signaling Technology, #9252, 1:1000 for WB)
ERK1/2 (Cell Signaling Technology, #4695,1:1000 for WB)
p38 (Cell Signaling Technology, #8690,1:1000 for WB)
phospho-JNK (Cell Signaling Technology, #4668, 1:1000 for WB)
phospho-ERK1/2 (Cell Signaling Technology, #9212, 1:1000 for WB)
phospho-p38 (Abcam, ab178867, 1:1000 for WB, 1:100 for IHC)
α-tubulin (Santa Cruz, sc-8035, 1:1000 for WB)
β-actin (Santa Cruz, sc-69879, 1:1000 for WB)
K5 (Santa Cruz, sc-80606, 1:100 for IHC)
K10 (Santa Cruz, sc-53251, 1:100 for IHC)
K17 (Santa Cruz, sc-393002, 1:100 for IHC)
GAPDH (Santa Cruz, sc-32233, 1:1000 for WB)
Ser/Thr antibody (Millipore, 05368, 1:1000 for WB)
Gli1 (Abcam, ab217326, 1:500 for WB, 1:100 for IHC, 1:50 for IP)
Gli2 (Abcam, ab26056, 1:500 for WB)
Gli3 (Abcam, ab69838, 1:500 for WB)
Smo (Abcam, ab236465, 1:1000 for WB)
Ki67 (Abcam, ab15580, 1:100 for IHC)
BLBP (Abcam, ab32423, 1:200 for IF)
Histone H3 (Beyotime Biotechnology, AF0009,1:500 for WB)
Flag (Beyotime Biotechnology, AF0036, 1:1000 for WB)
HA (Beyotime Biotechnology, AF2305, 1:1000 for WB, 1:50 for IP)
Pax6 (Hua-An Biotechnology ,ET1612, 1:100 for IF)
NeuN (Hua-An Biotechnology, ET1602, 1:100 for IF)
Calbindin (Hua-An Biotechnology, ET1702, 1:100 for IF)
Myc (Hua-An Biotechnology, R1208, 1:1000 for WB, 1:50 for IP)
Alexa-555 (Life Technology, S21381, 1:1000 for IF)
Alexa-488 (Life Technology, S32354, 1:1000 for IF)
Anti-rabbit IgG (Cell Signaling Technology, #7074, 1:10000 for WB)
Anti-mouse IgG (Cell Signaling Technology, #7076, 1:10000 for WB)
p-Ser937-GLI1 (Generation by GLS Biochemistry, 1:1000 for WB, 1:100 for IHC)

Validation

All antibodies were validated by western blotting of the respective antigen and the detection of the band of the expected size according to the statement reported on the manufacturer's websites.
Custom antibody against p-Ser937-Gli1 was validated by western blotting in GLI1-shRNA or neutralizing polypeptide treated 293T cells.

Eukaryotic cell lines

Policy information about cell lines and Sex and Gender in Research

Cell line source(s)

The mouse C3H10T1/2 cells and human HEK293T and Daoy cells used in the present study were obtained from National Collection of Authenticated Cell Cultures (Shanghai, China). The mouse MEF cells derived from pregnant mice at 14 to 16 days gestation.

Authentication

The cell lines used in the study were authenticated by National Collection of Authenticated Cell Cultures using STR profiling.

Mycoplasma contamination

We declare that all cell lines used in this research are mycoplasma negative by experimental detection.

Commonly misidentified lines
(See ICLAC register)

No commonly misidentified cell lines were used.

Animals and other research organisms

Policy information about studies involving animals; ARRIVE guidelines recommended for reporting animal research, and Sex and Gender in Research

Laboratory animals	Wild-type C57BL/6J mice were purchased from Shanghai SLAC Laboratory Animal Co. Ltd. (Shanghai, China). Smo-M2 and Smof/f mice were purchased from the Jackson Laboratory (Bar Harbor, ME) and generated as previously described 49. GFAP-Cre mice were gifted from prof. Chong Liu at Department of Pathology, Zhejiang University School of Medicine. Gli1(S941A)+/- and Gli1(S941E)+/- founders were generated by CRISPR/Cas9 at Shanghai Model Organisms Center, Inc. (Shanghai, China). All experimental animals were housed in a specific pathogen-free (SPF)-grade environment with room conditions of approximately 22-26℃ with 40-60% humidity, and a 12/12 hour light cycles. We used age- matched littermates of both sexes, aged between 2 and 24 weeks in this study. 2-month-old mice were used for breeding and 2- or 6-month-old male and female mice were used for experimental observations.
Wild animals	The study did not involve wild animals.
Reporting on sex	The study did not involve sex-based analysis.
Field-collected samples	The study did not involve samples collected from the field.
Ethics oversight	All animals were housed and bred at the Hangzhou City University School of Medicine Animal Care Facility according to the institutional guidelines for laboratory animals, and the protocol (No. 23004) was approved by the Institutional Animal Care and Use Committee of Hangzhou City University School of Medicine. The diameter of tumors in mice allowed by the Institutional Animal Care and Use Committee of Hangzhou City University School of Medicine is less than 20 mm, whereas the diameter of cerebellar tumors in mice in this study was approximately 5-8 mm.

Note that full information on the approval of the study protocol must also be provided in the manuscript.

Plants

Seed stocks	N/A
Novel plant genotypes	N/A
Authentication	N/A
